# Light‐Activated Molecules Targeting G‐Quadruplex Nucleic Acids

**DOI:** 10.1002/chem.202501545

**Published:** 2025-06-23

**Authors:** Marta Dudek, Clément Cabanetos, Marco Deiana

**Affiliations:** ^1^ Institute of Advanced Materials Faculty of Chemistry Wrocław University of Science and Technology Wyb. Wyspiańskiego 27 50–370 Wrocław Poland; ^2^ CNRS MOLTECH‐ANJOU SFR MATRIX Univ Angers F‐49000 Angers France

**Keywords:** cancer, G‐quadruplex, light‐activated therapies, photocaged ligands, photopharmacology, photosensitizers, photoswitches, precision therapy

## Abstract

Phototherapies harness light's spatial and temporal precision to noninvasively modulate biomolecular interactions, providing a powerful platform for precision oncology. This approach is particularly effective in targeting disease‐associated molecular structures such as G‐quadruplexes (G4s), noncanonical nucleic acid conformations found in oncogene promoters and telomeres. These unique structures can impede DNA polymerase progression along the duplex, triggering DNA damage that ultimately compromises genomic stability. Stabilizing G4s with tailored ligands is currently being explored as a promising anticancer strategy, as it may induce toxic DNA damage specifically in rapidly dividing cancer cells. Unlike conventional G4 ligands, which remain continuously active, newly developed light‐responsive molecules incorporate an OFF‐ON switching mechanism that allows for spatiotemporal control over G4 dynamics and phototherapeutic effects. This concept article reviews a diverse array of light‐responsive molecular tools, including photosensitizers (PSs), photocages, photochemically transformed ligands, and photoswitches, that selectively modulate G4‐interactive binding properties, thereby laying the foundation for a versatile photopharmacological platform. Additionally, the article highlights recent advancements in this rapidly evolving field and discusses the challenges that remain for clinical translation, underscoring the significant potential of G4‐targeted phototherapies to shape next‐generation cancer treatments.

## Graphical abstract

We shine a spotlight on the molecular recognition mechanisms and phototherapeutic potential of light‑activated compounds targeting G‑quadruplex nucleic acids, critically evaluating recent breakthroughs and charting the key challenges that must be overcome to drive this promising field forward.

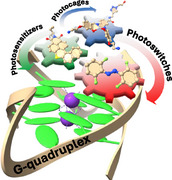

## 1 Introduction

In 2022, the Telomere‑to‑Telomere consortium achieved a landmark breakthrough by producing the first truly complete human genome assembly^[^ ^1^ ^]^ a contiguous, 3.05 gigabase‑pair sequence covering all 22 autosomes and the X chromosome. This pioneering work unveiled previously inaccessible regions and showed that nearly 54% of the assembly consists of repetitive elements.^[^ ^2^ ^]^ Moreover, kinetic analysis of single‑molecule real‑time (SMRT) sequencing data revealed that roughly 13% of the genome adopts noncanonical DNA conformations, including G4s.^[^ ^3^ ^]^

G4s are highly stable, four‐stranded structures formed within guanine(G)‐rich sequences during essential cellular processes like replication, transcription, and repair.^[^ ^4^, ^5^ ^]^ Structurally, G4s consist of stacked G tetrads stabilized by Hoogsteen hydrogen bonds and coordinated metal ions, predominantly potassium.^[^ ^6^ ^]^ Recent advances in sequencing technologies have identified approximately 700,000 G4 structures in the human genome,^[^ ^7^ ^]^ with notable enrichment at oncogene promoters, telomeric regions, replication origins, and genomic instability hotspots.^[^ ^5^, ^8^, ^9^ ^]^ These findings implicate G4 structures in critical cellular functions, including transcriptional control, replication stress responses, and DNA recombination.^[^ ^5^ ^]^

Under physiological conditions, G4s play vital roles in gene expression and telomere maintenance.^[^ ^8^, ^10^ ^]^ However, excessive or aberrant G4 formation can impede telomerase activity, stall DNA polymerases, and halt replication forks, ultimately triggering DNA damage responses.^[^ ^11^, ^12^, ^13^, ^14^ ^]^ Cancer cells, often characterized by elevated replication stress, oncogenic dysregulation, and compromised DNA damage repair pathways, exhibit impaired ability to resolve these G4 structures.^[^ ^9^, ^15^, ^16^ ^]^ Consequently, this vulnerability makes cancer cells particularly sensitive to therapeutic strategies designed to stabilize G4 structures, leading to enhanced genomic instability and cell death.^[^ ^6^, ^12^ ^]^

A prominent therapeutic approach involves small‐molecule ligands that selectively stabilize G4 structures, interfering with transcription and replication.^[^ ^11^, ^12^, ^17^ ^]^ Typically, these ligands possess planar heteroaromatic cores that engage G‐quartets via π–π stacking interactions, decorated by side‐chain modifications enhancing specificity through groove and loop interactions.^[^ ^4^ ^]^ Despite encouraging in vitro outcomes, clinical translation remains limited due to inadequate selectivity, as traditional ligands indiscriminately target G4s genome‐wide, resulting in significant off‐target toxicity.^[^ ^6^ ^]^

To address these limitations, researchers developed several innovative strategies to enhance G4 targeting specificity, including ligand‐peptide nucleic acid (PNA)^[^ ^18^ ^]^ and ligand‐oligonucleotide conjugates,^[^ ^19^ ^]^ duplex‐quadruplex hybrid structures,^[^ ^20^ ^]^ and PNA derivatives.^[^ ^21^ ^]^ Nevertheless, each approach carries intrinsic drawbacks: oligonucleotide conjugates are prone to rapid enzymatic degradation, and both duplex‐quadruplex hybrids and PNA‑based compounds exhibited only modest sequence specificity, which severely limited their application to test tube settings.

**Scheme 1:**
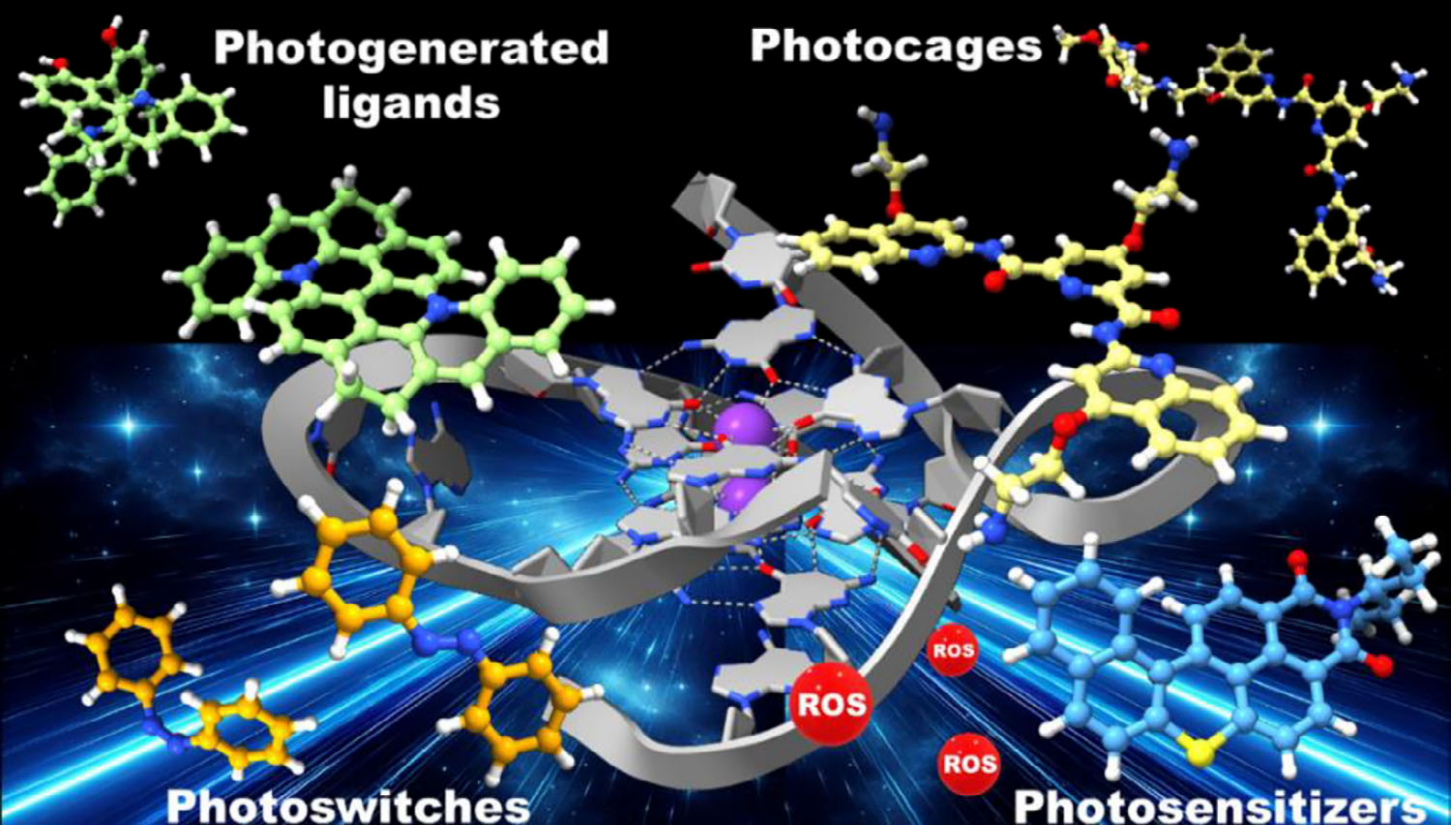
Schematic representation of the classes of light‑activated G4‑targeting compounds discussed in this concept.

Recently, light‐activated therapeutic approaches^[^ ^22^ ^]^ have emerged as an innovative solution to overcome these limitations, offering unprecedented spatiotemporal control over G4‐targeted drug activation,^[^ ^23^ ^]^ significantly reducing systemic toxicity. The general strategy of using light as a stimulus to activate a ligand is rooted in pioneering studies on photocontrollable DNA binders and photosensitizers (PSs) initially developed to target duplex DNA.^[^ ^24^ ^]^ Building upon this foundation, PSs,^[^ ^25^, ^26^, ^27^, ^28^, ^29^, ^30^, ^31^, ^32^, ^33^, ^34^ ^]^ photocaged ligands,^[^ ^35^, ^36^, ^37^, ^38^, ^39^, ^40^, ^41^ ^]^ photo‐transformed compounds,^[^ ^42^, ^43^, ^44^ ^]^ and photochemically responsive molecular switches^[^ ^45^, ^46^, ^47^, ^48^, ^49^, ^50^, ^51^, ^52^, ^53^, ^54^, ^55^, ^56^, ^57^, ^58^ ^]^ have been developed, enabling dynamic regulation of ligand–G4 interactions through controlled illumination (Scheme 1).

Critically, these photo‐responsive compounds exhibit either unidirectional or bidirectional switching behaviors upon light exposure. Unidirectional switches irreversibly convert from an inactive to an active form, providing sustained biological responses following illumination. This strategy is particularly beneficial in scenarios where prolonged activation is desirable. Conversely, bidirectional switches allow reversible toggling between active and inactive states, affording dynamic, real‐time modulation of therapeutic activity. This reversibility facilitates finely tuned, repeated dosing and adaptive treatment strategies, significantly enhancing therapeutic flexibility and reducing potential side effects.

By harnessing light as a finely tuned modulator, these cutting‐edge photo‐regulatory approaches pave the way for G4‐targeted interventions that minimize off‐target effects. As a result, light‐activated G4 ligands are poised to become essential building blocks for next‐generation personalized treatments. This concept article explores a variety of strategies for embedding optical control into G4‐binding compounds and highlights their transformative potential.

## 2 Light‐Activated Phototherapeutic Strategies: A Unidirectional Control Approach

### 2.1 Photosensitizers

Many oncogenes and cancer‐associated genes feature G‐rich promoter regions capable of folding into G4 structures.^[^ ^4^ ^]^ Due to G's low oxidation potential, these sequences are particularly susceptible to oxidative damage, especially under oxidative stress frequently encountered in cancer.^[^ ^59^, ^60^, ^61^ ^]^

**Scheme 2:**
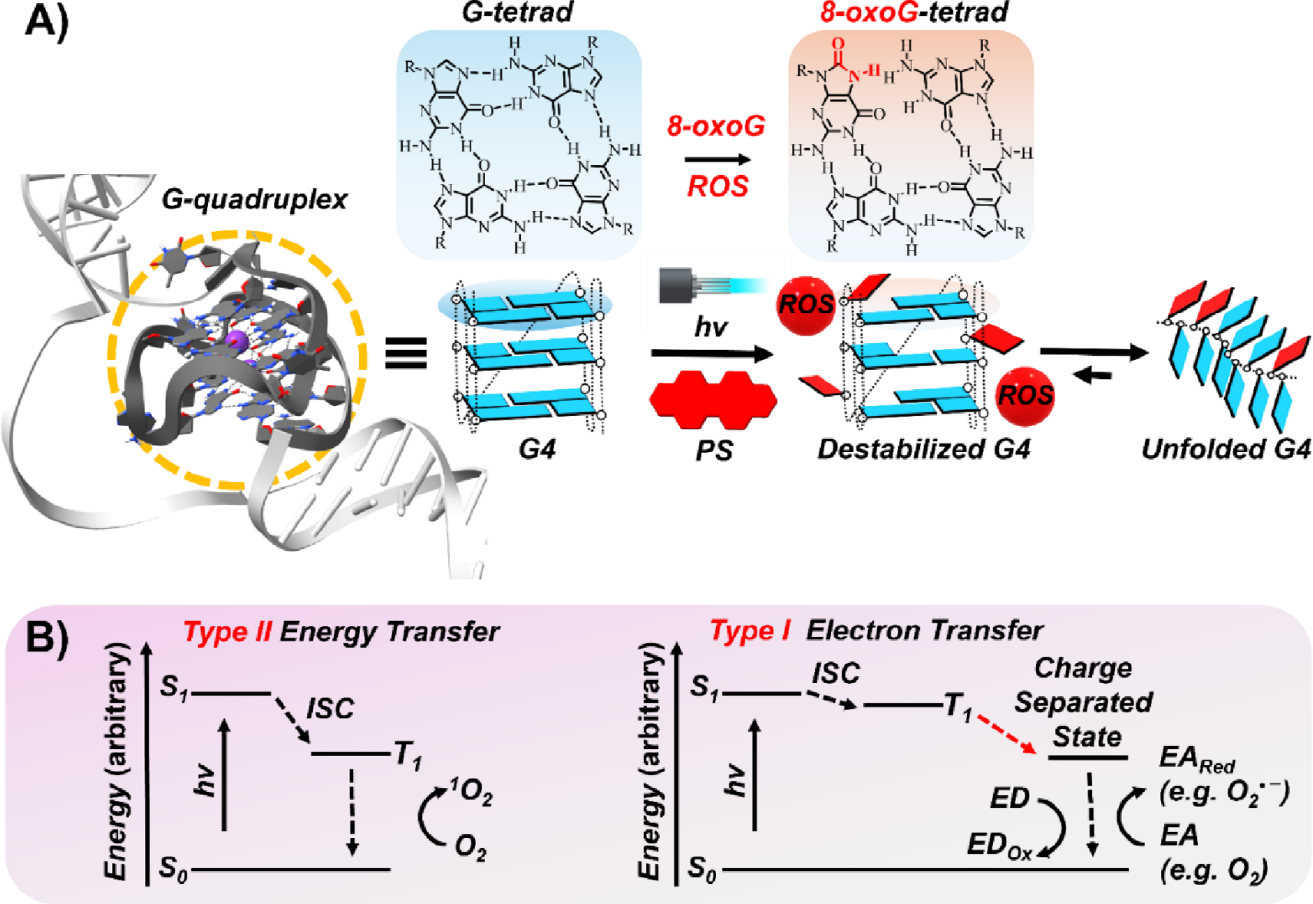
A) Schematic representation of a G4 DNA structure and the oxidative damage mediated by a PS. B) Type II and Type I photosensitization mechanisms. S_0_, S_1_, and T_1_ denote the singlet ground state, first excited singlet state, and first excited triplet state, respectively.

Reactive oxygen species (ROS), such as singlet oxygen (^1^O_2_), specifically oxidize Gs within G4 structures, generating lesions like 8‑oxo‑7,8‑dihydroguanine (8‐oxoG) (Scheme 2).^[^ ^62^ ^]^ The accumulation of 8‐oxoG destabilizes G4 structures, altering gene regulation.^[^ ^60^ ^]^ Photodynamic therapy (PDT) represents a promising approach for controlled ROS generation, requiring three critical components: a PS, molecular oxygen, and light.^[^ ^63^ ^]^ PDT achieves selectivity through spatiotemporal control of light activation and preferential PS accumulation in tumor tissues.

PSs generate ROS primarily through two mechanisms upon light irradiation: Type II energy transfer and Type I electron transfer (Scheme 2). In the Type II pathway, the excited triplet state of the PS undergoes a spin‐allowed energy transfer to ground‐state molecular oxygen (O_2_), a triplet diradical, converting it into electronically excited ^1^O_2_.^[^ ^64^ ^]^ In contrast, the Type I mechanism involves electron transfer reactions initiated by the excited PS.^[^ ^65^ ^]^ Generally, the excited PS is first reduced by an electron‐rich substrate (donor), such as nicotinamide adenine dinucleotide (NADH), forming a radical anion (PS^•−^).^[^ ^66^, ^67^ ^]^ This radical anion then acts as an electron donor, transferring an electron to O_2_ (acceptor) and generating superoxide anion radicals (O_2_^•−^).^[^ ^66^ ^]^ Subsequent redox transformations of O_2_^•−^, including dismutation to hydrogen peroxide (H_2_O_2_) and metal‐catalyzed reactions such as Fenton chemistry, can produce highly reactive hydroxyl radicals (OH^•^), contributing to oxidative damage.^[^ ^63^, ^68^ ^]^ The short lifetime and limited diffusion of these ROS confine oxidative damage to the vicinity of the activated PS, making the development of G4‐targeted PSs a significant advancement in cancer therapeutics.

An ideal G4‐specific PS should exhibit negligible dark toxicity, bind selectively to G4 structures without excessively stabilizing them, thus preserving natural DNA replication and transcription processes, and become therapeutically active upon illumination to induce structure‐specific oxidative DNA damage.

**Figure 1:**
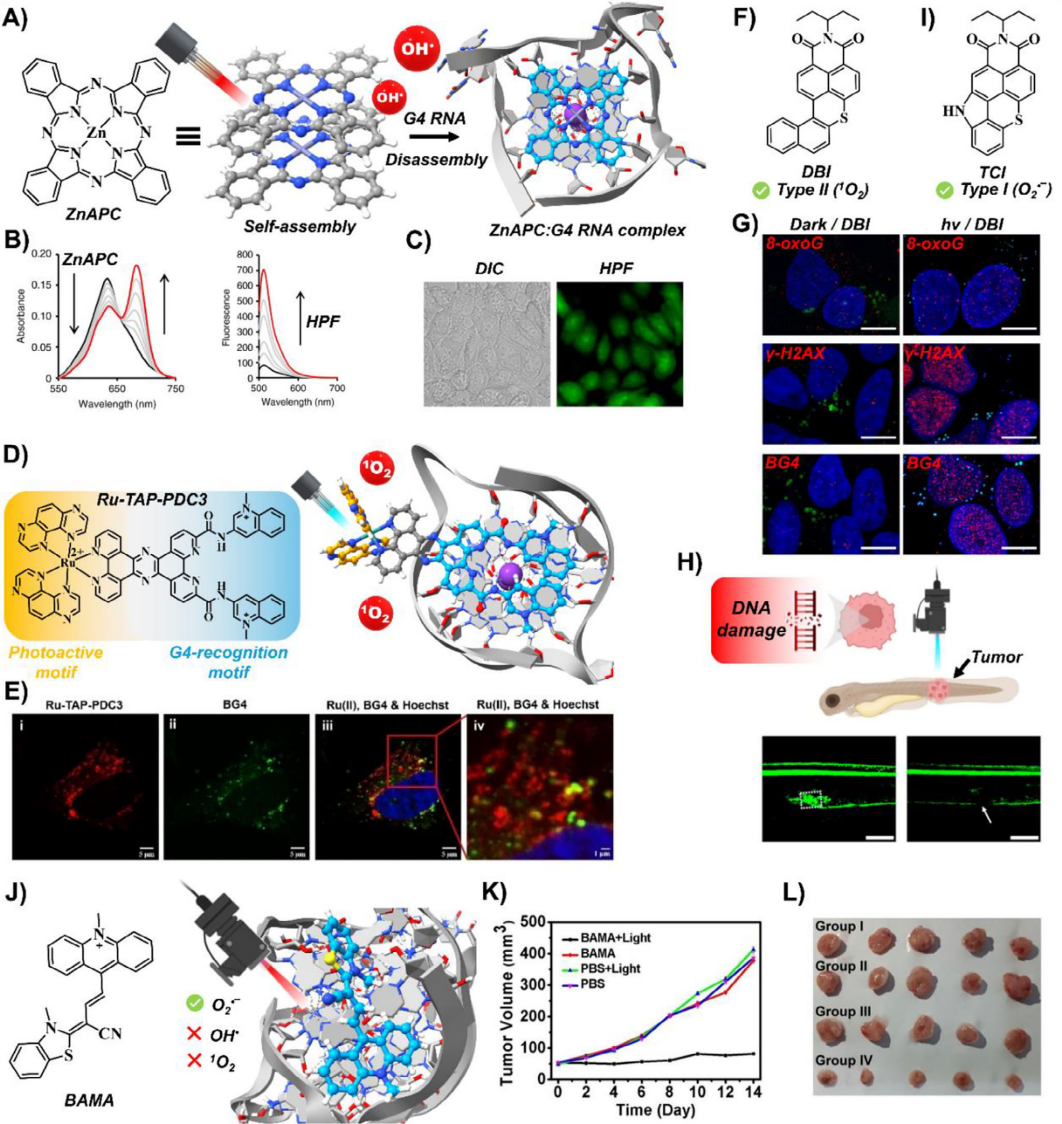
A) Chemical structure of **ZnAPC** and its binding mode to an RNA G‑quadruplex (PDB ID: 7SXP). B) Vis‐NIR absorption spectra of **ZnAPC** showing aggregate‐to‐monomer transition upon complexation with the RNA G4. Reproduced with permission from ref. [28]. Copyright 2018 Springer Nature C) Fluorescence imaging of OH^•^‑specific HPF in MCF‑7 cells following photoactivation of **ZnAPC**. Reproduced with permission from ref. [28]. Copyright 2018 Springer Nature. D) Chemical structure of **Ru‑TAP‑PDC3** and its binding to a DNA G‑quadruplex (PDB ID: 1K8P). E) Confocal images demonstrating co‑localization of **Ru‑TAP‑PDC3** with BG4 in mitochondria. Reproduced with permission from ref. [29]. Copyright 2024 Wiley‐VC. F) Chemical structure of **DBI**. G) **DBI**‑induced DNA damage upon irradiation, evidenced by increased nuclear foci for 8‑oxoG, γ‐H2AX and BG4. Reproduced with permission from ref. [25]. Copyright 2023 Oxford University Press. H) In vivo phototherapeutic ablation of rhabdomyosarcoma in zebrafish following **DBI** treatment and light exposure. The withe arrow indicates the tumor area. Reproduced with permission from ref. [31]. Copyright 2025 American Chemical Society. I) Chemical structure of **TCI**. J) Chemical structure of **BAMA** and its exclusive O_2_^•−^ generation upon complexation with an RNA G4 (PDB ID: 2M18). K) Tumor growth curves of 4T1‑bearing mice under various treatment regimens. Reproduced with permission from ref. [26]. Copyright 2023 Wiley‐VCH. L) Photographs of excised tumors at study end, illustrating treatment efficacy. Reproduced with permission from ref. [26]. Copyright 2023 Wiley‐VCH.

G4‐specific PSs are categorized into metal‐based and metal‐free compounds. Incorporating heavy atoms such as halogens (Br, I) or transition metals significantly enhances intersystem crossing (ISC) efficiency through heavy‐atom‐induced spin‐orbit coupling (SOC), thereby boosting their photosensitizing ability.^[^ ^69^ ^]^ For instance, Kawauchi et al. developed an anionic zinc phthalocyanine (**ZnAPC**) PS that transitions from aggregated forms to active monomers upon binding specifically to a G4 RNA structure found in the 5´‐untranslated region (UTR) of *NRAS* mRNA.^[^ ^28^ ^]^ **ZnAPC** was efficiently internalized by MCF‐7 breast cancer cells, generated OH^•^ radicals upon irradiation at 630 nm, and selectively downregulated *NRAS* expression, ultimately inducing cell death (Figure 1A–C).

Vilar and colleagues designed Pt(II) complexes that selectively bind G4s, turn on fluorescence upon G4 recognition, and exhibit minimal dark cytotoxicity, with some derivatives localizing to G4‑rich nucleoli.^[^ ^33^ ^]^ Under 457 nm irradiation, these complexes generate ^1^O_2_ and/or O_2_^•−^ anion radicals and achieve half‑maximal inhibitory concentration (IC_50_) values as low as 294 nM in HeLa cervical cancer cells.

Keyes and co‑workers developed **Ru‑TAP‑PDC3**, a photoactive Ru(II) complex conjugated to the high‑affinity G4 ligand Phen‑DC3,^[^ ^70^ ^]^ to selectively target mitochondrial G4s (Figure 1D–E).^[^ ^29^ ^]^ Mitochondrial (mt) DNA is a circular ∼16.5 kb genome encoding 37 genes essential for oxidative phosphorylation and containing over 200 putative G4‑forming sites; its high K^+^ concentration (150–180 mM) promotes G4 folding, and, lacking histones and robust repair pathways, mtDNA is especially susceptible to G4‑targeting agents.^[^ ^71^ ^]^

In HeLa cells, chosen for their mtDNA‑rich mitochondria, **Ru‑TAP‑PDC3** localization was confirmed by co‑staining with BioTracker 488 and the G4‑specific antibody BG4.^[^ ^15^ ^]^ The conjugate showed negligible dark toxicity (IC_50_ > 200 µM) but, upon 470 nm irradiation, triggered potent apoptosis‑mediated cell death (IC_50_ ∼ 1–3 µM), even under hypoxia. Its efficacy, however, was markedly reduced in 3D HeLa spheroids, likely due to increased dark toxicity in that model.

Despite their promise, metal‑based PSs often suffer from intrinsic dark toxicity, complex syntheses, and high production costs.^[^ ^72^ ^]^ This has driven interest in metal‑free alternatives. Our groups advanced this area by developing benzothioxanthene imide‑based PSs,^[^ ^73^ ^]^ most notably dibenzothioxanthene imide (**DBI**) dyes^[^ ^25^, ^74^ ^]^ (Figure 1F). Through a twisting‑induced ISC mechanism, the sulfur‑substituted **DBI** achieved near‑quantitative ^1^O_2_ generation and displayed exceptional targeting selectivity for G4 over duplex DNA.^[^ ^25^ ^]^ **DBI** exhibited negligible dark toxicity in HeLa and MCF‑7 cells up to 25 µM. However, upon 470 nm irradiation, it induced potent phototoxicity (IC_50_ ∼ 20 nM) and DNA damage (Figure 1G), achieving a phototherapeutic index, defined as the ratio between IC_50_ in dark and light conditions, of >1000 in 2D monolayers, and a markedly high index also in pancreatic 3D tumor organoids. In vivo validation in a zebrafish rhabdomyosarcoma model confirmed **DBI**’s remarkable phototherapeutic efficacy, ablating cancer cells only upon light activation (Figure 1H).^[^ ^31^ ^]^

Building on this strategy, our groups developed a thiochromenocarbazole imide (**TCI**) dye as an organelle‑targeted PS for mitochondria and the endoplasmic reticulum (ER), which predominantly generates O_2_^•−^ anion radicals (Figure 1I).^[^ ^30^ ^]^ O_2_^•−^ production was markedly enhanced upon binding the oncogenic G4 motif from the *MYC* gene, highlighting the pivotal role of G4 structures in amplifying photosensitization.^[^ ^61^ ^]^

Complementing these insights, Chen et al. developed a benzothiazolium‐acetonitrile‐methylacridinium conjugate (**BAMA**), exhibiting selective fluorescence activation and potent O_2_^•−^ anion radical generation upon targeting G4 RNA structures (Figure 1J).^[^ ^26^ ^]^ **BAMA** demonstrated impressive phototherapeutic efficacy at 660 nm under normoxic and hypoxic conditions, despite limited selectivity toward cancer versus normal cells. Nonetheless, in vivo studies using murine 4T1 breast cancer models demonstrated significant tumor growth inhibition (∼80%) following irradiation (Figure 1K–L).

Ma and colleagues designed **TPAL**, a water‐soluble, amphiphilic aggregation‐induced emission (AIE)‐active probe built on a tetraphenylethylene core, lactose ligands for asialoglycoprotein receptor (ASGPR)‐mediated uptake, and a pyridinium mitochondrial‐targeting unit.^[^ ^34^ ^]^ In buffer solution, **TPAL** self‐assembled, but binding mitochondrial G4s triggered a monomerization into a bright emissive complex that generated ^1^O_2_ under white light. It accumulated in mitochondria with negligible dark toxicity and showed stronger phototherapeutic effects in HepG2 liver cancer cells than in normal LO2 cells.

Collectively, these studies underscore the transformative potential of G4‐specific PSs, highlighting both metal‐based and metal‐free approaches as powerful tools in targeted anticancer phototherapy.

### 2.2 Photocaged and Photo‐Transformed Compounds

**Figure 2:**
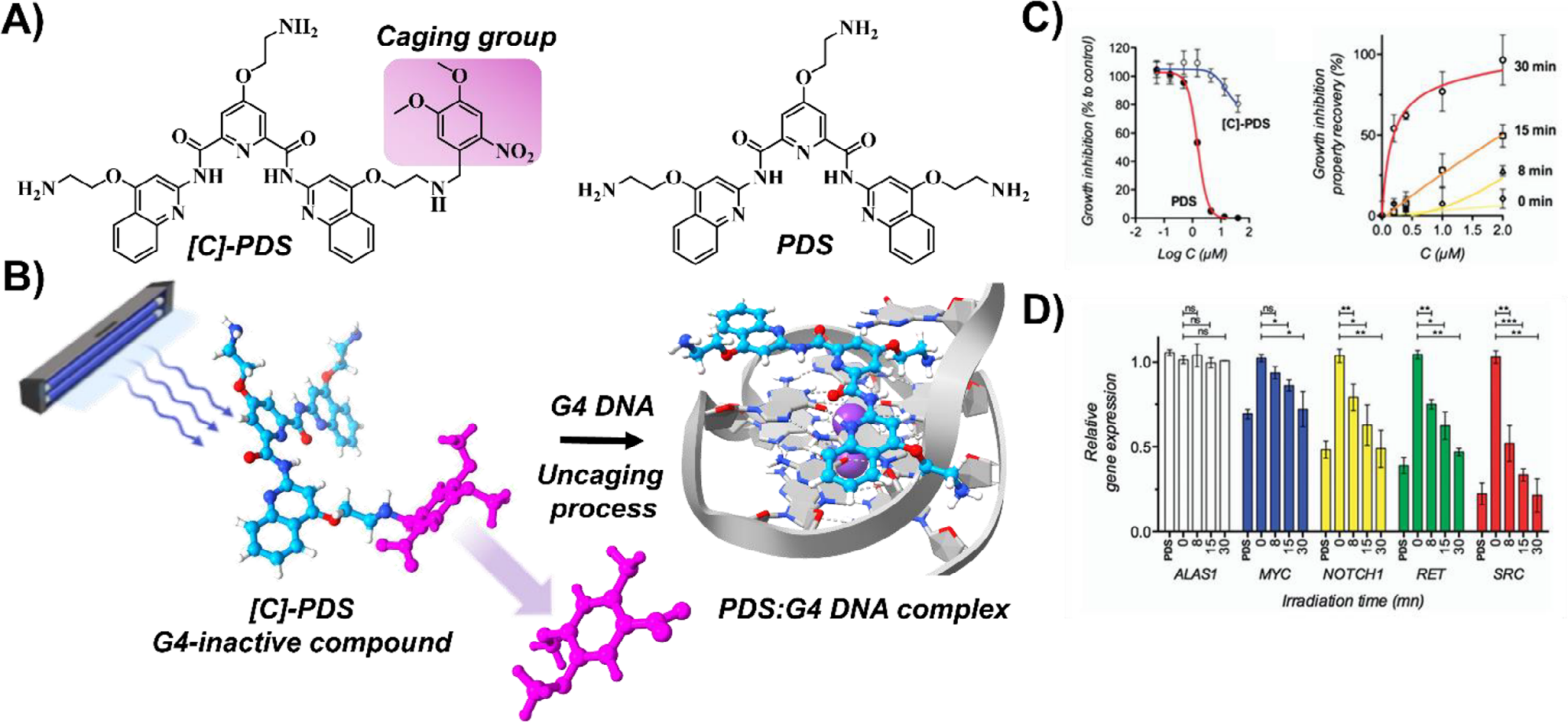
A–B) Chemical structures of caged **[C]‑PDS** and **PDS** and the G4‑interactive binding mechanism (PDB ID: 2L7V). C) Growth‑inhibition properties of **[C]‑PDS** and **PDS** at different concentrations in MRC5‑SV40 human fibroblast cells (left panel) and comparison of the effect of **[C]‑PDS** at varying concentrations and irradiation times with that of 2 µM **PDS** (right panel). Reproduced with permission from ref. [36]. Copyright 2013 Royal Society of Chemistry. D) Light‑mediated downregulation of mRNAs of G4‑containing genes in MRC5‑SV40 cells treated with 2 µM **[C]‑PDS** and irradiated at 365 nm for 0, 8, 15, or 30 minutes, or treated with 2 µM **PDS**. Reproduced with permission from ref. [36]. Copyright 2013 Royal Society of Chemistry.

An innovative way to achieve spatial and temporal control of molecular interactions is to “cage” bioactive compounds with photo‐protecting groups (Figure 2A–B).^[^ ^75^ ^]^ For example, classic G4 binders such as telomestatin^[^ ^35^ ^]^ and pyridostatin (**PDS**)^[^ ^36^ ^]^ were inactivated by masking their binding sites with nitroveratryl groups. In this caged form, they remained biologically dormant and could not engage G4 structures. Upon 365 nm UV irradiation, the nitroveratryl groups were cleanly cleaved in a photo‐deprotection step, fully restoring G4 binding to levels matching the native ligands. This uncaging not only preserved the compounds’ original binding affinity but also enabled light‑triggered telomerase inhibition,^[^ ^35^ ^]^ photocontrolled cytotoxicity^[^ ^36^ ^]^ (Figure 2C), downregulation of G4‑driven oncogene transcription^[^ ^36^ ^]^ (Figure 2D), and spatiotemporal visualization of G4 DNA in living cells.^[^ ^37^ ^]^

**Figure 3:**
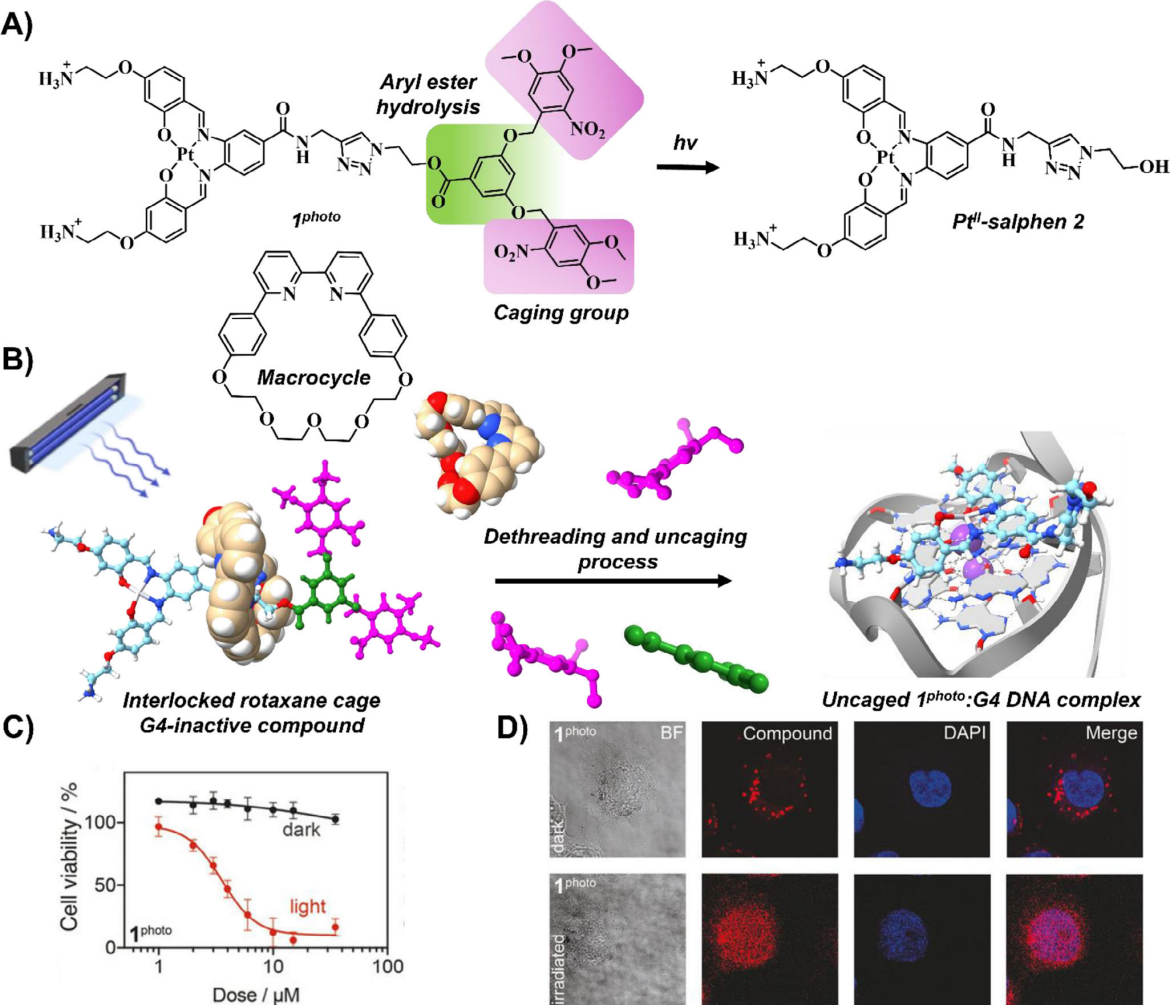
A) Chemical structures of **1^photo^**, the macrocycle, and the uncaged Pt^II^‑salphen **2**. B) Proposed mechanism of action of **1^photo^** for light‑triggered G4 interactions (PDB ID: 2L7V). C–D) Cell‑viability assay C) and fixed‑cell imaging D) of **1^photo^** in U2OS cells under dark and light conditions. Reproduced with permission from ref. [38]. Copyright 2021 Wiley‐VC.

Building on this concept, Vilar's group engineered a sophisticated system by incorporating photolabile masking groups within a rotaxane architecture (Figure 3A).^[^ ^38^ ^]^ In their design, a Pt^II^‐salphen complex (**2**), renowned for its G4‐interactive binding properties, was integrated with a macrocycle and two nitroveratryl groups, yielding a compound (**1^photo^**) that featured a two‐stage activation process (Figure 3B). Upon irradiation at 365 nm, the subsequent cleavage of the nitroveratryl groups triggered the dethreading of the macrocycle, thereby restoring the complex's G4‐binding capability. This activation not only enhanced cellular uptake and facilitated nuclear localization but also demonstrated promising biological effects (Figure 3C–D). Photocytotoxicity studies in U2OS osteosarcoma cells showed that the caged compound exhibited minimal toxicity in its inactive state, whereas light activation restored its biological activity, resulting in robust cytotoxicity with an IC_50_ of 3.8 µM.

In contrast to the examples described above, uncaged ligands can promote the subsequent unfolding of G4 structures. For instance, bulky ruthenium complexes equipped with photolabile ligands underwent a ligand exchange reaction upon light exposure, in which the labile moiety was replaced by water to form a reactive aquo complex.^[^ ^39^, ^40^ ^]^ This transformation preferentially targeted accessible Gs, leading to metalation and subsequent disruption of G4 folding, a phenomenon that correlated with increased expression of the *c‐MYC* gene following irradiation.^[^ ^39^ ^]^ Additionally, photocleavable linkers were employed to modulate the topological states of a G4‐forming aptamer. Barthélémy and colleagues conjugated a lipid to the thrombin‐binding aptamer (TBA) via an O‑nitrobenzyl linker.^[^ ^41^ ^]^ Under physiological conditions, this conjugation enforced an inactive, parallel conformation; however, exposure to 350 nm light cleaved the linker, thereby restoring the native antiparallel structure required for efficient protein interaction.

A further approach involved molecules designed to undergo significant structural reorganization upon light exposure. Galan's group explored stiff‐stilbene derivatives decorated with either a rigid methylpyridinium moiety or a more flexible amine side chain.^[^ ^42^, ^43^ ^]^ The derivative with the rigid substitution was particularly notable, as it unfolded the telomeric G4 structure under sodium‐rich conditions, a process that could be reversed by photooxidation to form a ketone upon 400 nm light irradiation.^[^ ^42^ ^]^ Although repeated cycles of folding and unfolding required periodic ligand replenishment, this approach offered dynamic control over G4 conformational states.^[^ ^42^ ^]^

**Figure 4:**
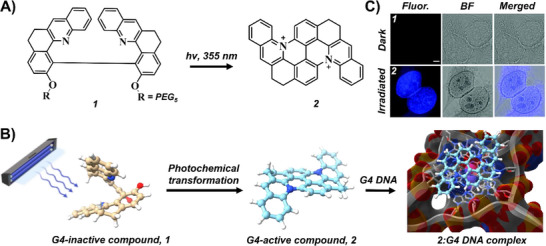
A–B) Chemical structures of compounds **1** and **2** and their photochemical transformation mechanisms targeting G4 structures (PDB ID: 1K8P). C) *In cellulo* photoconversion of compound **1** to compound **2** in fixed HeLa cells. Reproduced with permission from ref. [44]. Copyright 2021 Royal Society of Chemistry.

Independently, our groups demonstrated the selective photocyclization of a water‐soluble bisquinoline derivative (**1**) into a (diazonia)tetrabenzo naphthacene derivative (**2**), a π‑extended dicationic chromophore that retained excellent solubility in physiological media (Figure 4A–B).^[^ ^44^ ^]^ Triggered at 355 nm, this conversion not only activated its G4‑binding and stabilizing capabilities, as confirmed by high‑resolution DNA polymerase stop assays, but also enabled efficient *in cellulo* photoconversion with preferential nuclear localization in HeLa cells (Figure 4C). Notably, this compound generated ^1^O_2_ with a remarkable efficiency of 55%, thereby adding another layer of therapeutic potential.^[^ ^76^ ^]^

## 3 Light‐Activated Phototherapeutic Strategies: A Bidirectional Control Approach

A complementary approach to remotely controlling G4 structure and function involves incorporating molecular switches, molecules that possess at least two isomeric forms that are interconverted by light irradiation.^[^ ^77^ ^]^ Two primary mechanisms drive the photoisomerization process: a *trans*‐to‐*cis* isomerization, as observed in azobenzene, stiff‐stilbene, and similar systems; and a 6π electrocyclization of a triene system, as seen in dithienylethene derivatives.^[^ ^78^ ^]^ In addition, some systems, such as spiropyrans, exhibit a mixed mechanism that combines aspects of both processes.^[^ ^79^ ^]^ Light‐induced isomerization alters key properties, including polarity and geometry, thereby enabling control over the behavior of each isomer in the context of G4 DNA targeting and modulation. Ideally, one isomer is envisioned to be G4‐active while the other remains G4‐inactive. Although this optimal scenario is not yet fully realized, ongoing efforts aim to develop systems in which the isomer‐dependent response is functionally distinguishable as active or inactive.

Pioneering studies by Zhou and co‐workers over a decade ago laid the groundwork for this approach.^[^ ^45^, ^46^ ^]^ They designed an azobenzene derivative modified with N‐methylated piperidine that underwent isomerization under both UV and visible light. This switching process achieved a favorable isomer ratio in the *cis*‐rich photostationary state (PSS). In their system, the incorporation of the *trans* isomer into the telomeric DNA sequence promoted the formation of G4 structures, whereas UV irradiation induced *trans*‐to‐*cis* isomerization that led to G4 DNA unfolding.^[^ ^45^ ^]^ However, subsequent investigations revealed that under physiological conditions, a complete unfolding effect was not achievable. Instead, light provided reversible control over the topological changes in telomeric G4 DNA, shifting it from a hybrid to an antiparallel conformation.^[^ ^46^ ^]^

A few years later, Zhou's group extended their work by employing photoswitches to influence enzymatic reactions.^[^ ^48^, ^58^ ^]^ They demonstrated the potential of these systems to regulate thrombin activity by modulating the conformation of a DNA‐based inhibitor. This inhibitor system comprised a central telomeric G4‐forming sequence that functioned as a regulatory element, in tandem with two TBAs that cooperated to inhibit fibrinogen binding to thrombin. The geometrical state of the photoswitch, regulated by UV and visible light, determined whether the telomeric G4 element folded or unfolded, thereby enabling reversible control over the thrombin‐fibrinogen interaction.^[^ ^58^ ^]^

**Figure 5:**
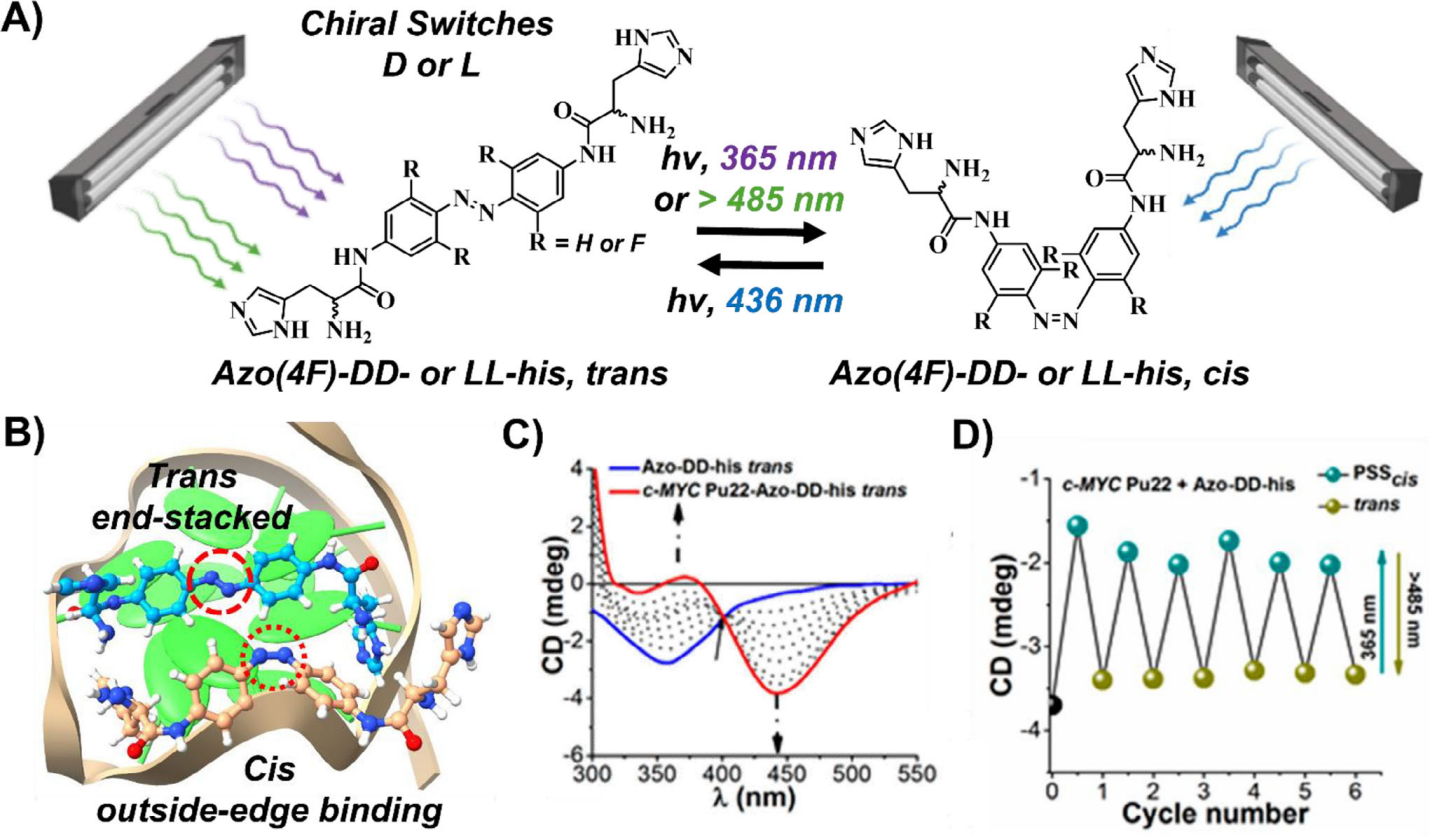
A–B) Chemical structures of the chiral photoswitches used to control G4‑interactive binding interactions (PDB ID: 2L7V). C–D) CD spectral changes upon complexation of **Azo‑DD‑his** in its *trans* configuration with incremental addition of *c‑MYC* G4, and photomodulation of chiroptical activity upon isomerization of the switch. Reproduced with permission from ref. [50]. Copyright 2021 American Chemical Society.

Concurrently, our research groups advanced the understanding of chiral recognition of azobenzene derivatives modified with chiral substituents, specifically L‐ and D‐amino acids (histidine or lysine), for the selective targeting of left‐ and right‐handed G4 structures (Figure 5A).^[^ ^49^, ^50^ ^]^ We examined both conventional azobenzene and tetra‐*ortho*‐fluoroazobenzene derivatives, noting distinct features such as the thermal stability of the *cis* isomer and the specific wavelengths required for isomerization (in the UV‐visible and visible‐visible ranges). Our studies involving chiral azobenzene switches functionalized with histidine units (**Azo(4F)‐DD‐his** or **Azo(4F)‐LL‐his**) revealed that right‐handed G4 structures were stabilized by both the *trans* isomer and the *cis*‐rich PSS mixture, although there was a more pronounced preference for stabilization by the *trans* form, while left‐handed G4 structures were more effectively stabilized by the *cis*‐rich mixture.^[^ ^50^ ^]^ Furthermore, these complex binding interactions gave rise to distinct coordination mechanisms and chiroptically active systems, featuring induced circular dichroism bands whose intensities could be dynamically tuned via light‑controlled switching (Figure 5B–D).^[^ ^49^, ^50^ ^]^

**Figure 6:**
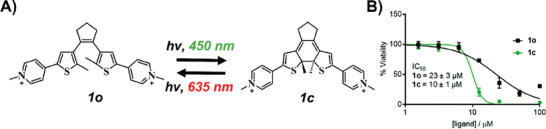
A–B) Chemical structure of compound **1** in both its open and closed forms and its effects on HeLa cell viability. Reproduced with permission from ref. [51]. Copyright 2020 Royal Society of Chemistry.

Recognizing that these early examples had been demonstrated in controlled test‐tube settings, researchers questioned whether such properties could be translated into biological systems. To investigate this, Galan's group examined dithienylethene derivatives, specifically photoisomers labeled **1o** and **1c**, that were switchable with visible light in both directions (450 nm and 635 nm) (Figure 6A).^[^ ^51^ ^]^ Using biophysical methods, they evaluated whether the isomers exhibited distinct responses toward G4 motifs. Although the differences in binding affinities and thermal stability between the isomers were limited, the two isomers adopted distinct binding modes that could be toggled on demand by light. Toxicity assays performed in HeLa cells revealed a two‐fold difference in toxicity between the isomers (Figure 6B). Further studies demonstrated the antiparasitic activity of a family of UV‐switched dithienylethene derivative isomers (including **1o** and **1c**) against trypomastigotes, the active bloodstream form of the protozoan *Trypanosoma cruzi* (*T. cruzi*) SOL strain, with a selectivity index, defined as the ratio between IC_50(MRC‐5)_ and IC_50(_ *_T. cruzi_*_)_, of approximately 41 over the MRC5 control cell line.^[^ ^52^ ^]^ Localization studies showed that compound **1o** accumulated within both the nucleus and kinetoplast, suggesting that the G4 ligand **1o** had the potential to reach and target G4 DNA within the parasite's genome. In a related study on antiparasitic activity using azobenzene‐based G4 ligands (tested only in the *trans* isomer form), high efficacy against *Trypanosoma brucei* (*T. brucei*) was reported, with an IC_50_ of approximately 0.7 nM and a selectivity index, defined as the ratio between IC_50(MRC‐5)_ and IC_50(_ *_T. brucei_*_)_, of around 2286 over MRC‐5 cells.^[^ ^53^ ^]^ The authors cautioned, however, that differences in cellular uptake, cell cycle rates, or nuclear membrane composition might have contributed to the exceptional selectivity observed in parasitic cells compared to mammalian cells.

Our groups further synthesized an *ortho*‐fluoroazobenzene derivative, **Py‐Azo4F‐3N**, which was distinguished by the high thermal stability of its *cis* isomer and efficient switching under visible light.^[^ ^54^ ^]^ Biophysical analysis revealed that while the *trans* isomer effectively stabilized antiparallel and hybrid G4 structures, the *cis*‐rich mixture did not. Cytotoxicity assays in HeLa cells and U2OS cells showed a twofold difference in toxicity (with IC_50(_ *_trans)_* ∼ 6 µM versus IC_50(_ *_cis‐rich PSS_*_)_ ∼ 13 µM), indicating that the biological effect varied depending on the photoswitch's conformation.

Despite mounting evidence on photoregulative processes in G4 targeting, several critical questions remained unresolved. In particular, it was unclear whether photoswitch‐induced cytotoxicity could be controlled within cells and directly correlated with changes in the G4‐folding landscape, leaving the connection to G4 regulation uncertain.

**Figure 7:**
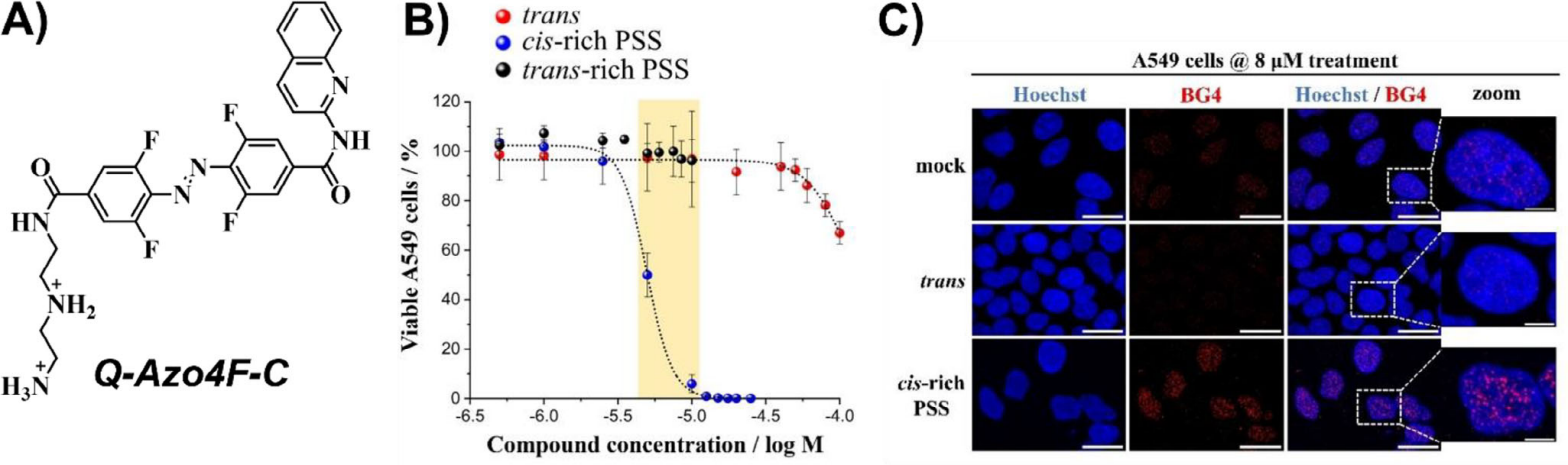
A) Chemical structure of **Q‑Azo4F‑C** in its *trans* form. B) Cell‑viability assays in A549 cells treated with **Q‑Azo4F‑C** in *trans* or *cis*‑rich PSS, and *in cellulo cis*‑to‑*trans* isomerization. Reproduced with permission from ref. [55]. Copyright 2025 Wiley‐VC. C) Confocal images for the immunodetection of endogenous G4 structures using BG4 with **Q‑Azo4F‑C** in *trans* or *cis*‑rich PSS. Reproduced with permission from ref. [55]. Copyright 2025 Wiley‐VC.

New insights emerged from our groups, who synthesized an *ortho*‐fluoroazobenzene derivative (**Q‐Azo4F‐C**) that was reversibly switchable with visible light (436 nm and ≥550 nm) and exhibited distinct coordination modes toward G4 structures (Figure 7A).^[^ ^55^ ^]^ We demonstrated that the *cis*‐rich PSS of **Q‐Azo4F‐C** was highly toxic to cancer cell lines, for example, lung adenocarcinoma A549 cells showed an IC_50(_ *_cis_*_‐rich PSS)_ of 4.8 µM, whereas the *trans* isomer produced negligible effects (IC_50(_ *_trans_*_)_ > 100 µM) (Figure 7B). Furthermore, we remotely controlled cytotoxicity through interconversion of the switch's state, with nearly complete photorecovery observed, depending on the cell line. Immunofluorescence studies using the BG4 antibody revealed that cells treated with the *trans* isomer displayed fewer BG4 foci, suggesting an unfolding of G4 structures mediated by the intercalative binding process, while treatment with the *cis*‐rich mixture resulted in increased nuclear BG4 foci, indicating G4 stabilization via outside‐edge binding (Figure 7C). Ultimately, *in cellulo* validation confirmed that *cis*‐to‐*trans* isomerization could regulate G4 folding dynamics.

**Figure 8:**
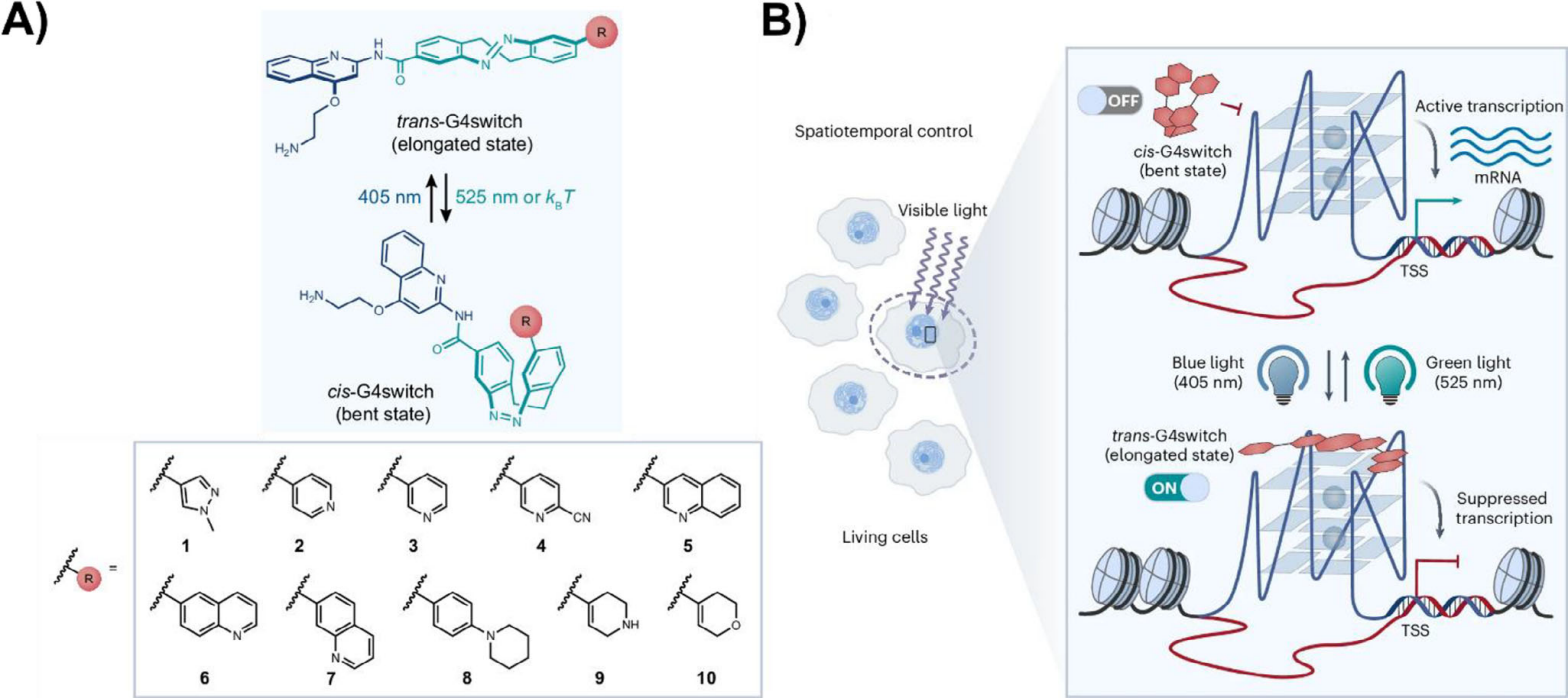
A) Molecular design of the diazocine compounds. Reproduced with permission from ref. [56]. Copyright 2025 Springer Nature. B) Schematic diagram illustrating the concept of optically controlling gene expression using a photoswitchable G4 ligand. Reproduced with permission from ref. [56]. Copyright 2025 Springer Nature.

Groundbreaking work by Balasubramanian and co‑workers further solidified the potential of molecular photoswitches as powerful tools for the reversible manipulation of G4 structures and functions in living cells.^[^ ^56^ ^]^ They developed cyclic azobenzene derivatives (diazocines) that were switchable exclusively by visible light. Among these, compound **9** exhibited outstanding activity, displaying distinct G4‑binding properties depending on its isomeric state: the *trans* isomer acted as a G4‑active binder, while the *cis* isomer remained G4‑inactive (Figure 8A). To verify G4‑selective and light‑dependent binding in a cellular context, a chemical mapping (Chem‑map) methodology was employed to track the interactions of the photoswitch with G4s in U2OS cells. For these experiments, a biotinylated variant of compound **9** was synthesized, retaining outstanding G4‑interactive binding properties similar to its unmodified precursor. As expected, the *trans* state of the switch bound with much higher prevalence to folded genomic G4 structures compared to the *cis* isomer. The ability of compound **9** to photomodulate gene expression in live U2OS cells was investigated by quantifying immediate changes in transcription using a nascent RNA detection method called SLAM‑seq. The *trans* isomer significantly downregulated transcriptional activity in 311 genes, whereas the *cis* form affected only 61 genes (Figure 8B). Notably, in situ photoswitching experiments revealed that gene expression changes induced by the *trans* isomer could be reversed by alternating cycles of light‑induced isomerization. These findings provide compelling evidence that azobenzene derivatives can be used to reversibly control gene expression via light‑mediated G4 targeting.

A separate study building on photoisomerization had integrated this approach into CRISPR technology to achieve control over gene editing and expression.^[^ ^57^ ^]^ In that work, a dicationic azobenzene derivative (**AZD^++^**) was embedded within a G4‑modified guide RNA (GqRNA), creating a light‑responsive complex. The isomeric state of **AZD^++^**, which could be reversibly switched by UV and visible light, toggled the CRISPR‐Cas9 system between active and inactive states. In its *trans* form, **AZD^++^** stabilized the G4 structure, inhibited R‑loop formation, and prevented DNA cleavage. Upon light‑induced isomerization to the *cis* form, the GqRNA unfolded and restored CRISPR activity, enabling genome editing. This approach was first validated in vitro and was subsequently applied in human cells, demonstrating tunable control over CRISPR function with minimal background activity.

Collectively, these studies contributed critical insights into the design, function, and potential biomedical applications of photoresponsive molecular switches for G4 DNA modulation.

## 4 Summary and Outlook

Interdisciplinary efforts at the chemistry–biology interface have firmly established G4 structures as pivotal regulators of DNA replication, transcription, telomere maintenance, and overall genome integrity.^[^ ^4^, ^5^, ^8^ ^]^ Despite medicinal‑chemistry campaigns yielding over a thousand small‑molecule G4 ligands,^[^ ^80^ ^]^ only a handful have progressed to clinical evaluation,^[^ ^81^ ^]^ underscoring the urgent need for next‑generation designs that integrate molecular recognition with robust functional efficacy. Photoresponsive G4 ligands promise unparalleled spatial and temporal precision: by tuning the irradiation wavelength, one can control exactly when, and in which subcellular compartment, a ligand binds or induces cytotoxicity. Although elegant proof‑of‑concept studies in cells have validated these approaches, several critical barriers must be overcome before they can advance toward patient‑ready phototherapeutics. One major limitation is the insufficient correlation between in vitro activity and in vivo efficacy. In many cases, it remains unclear whether the observed cytotoxic effects in cells are directly mediated by ligand‐G4 interactions or result from off‐target damage, such as general oxidative stress or nonspecific DNA binding. To resolve this, more sophisticated analyses, such as cell cycle profiling, transcriptomic studies, and G4‐specific chromatin immunoprecipitation, are essential to elucidate the precise mode of action. Furthermore, while some G4 ligands have shown encouraging antitumor effects in animal models, translation to clinical settings remains scarce. As of now, only a few G4‐targeting agents (e.g., CX‐5461)^[^ ^14^, ^82^ ^]^ have entered clinical trials, often hindered by poor pharmacokinetics, limited bioavailability, or off‐target toxicity.^[^ ^81^, ^83^ ^]^ The incorporation of light‐activated control in next‐generation ligands offers a promising avenue to overcome these barriers by enhancing selectivity and minimizing systemic side effects. To truly unlock the clinical potential of G4‐targeted phototherapeutics, a series of interdisciplinary challenges must be addressed. Below, we outline five key research directions that we believe are essential to advance this emerging field:

1. *G4 RNA*: To date, most photopharmacological strategies have focused on targeting G4 DNA structures within nuclear or mitochondrial compartments to modulate gene expression or induce localized DNA damage. This narrow focus is somewhat surprising, given that G4 RNA structures, typically adopting parallel topologies and exhibiting superior thermodynamic stability, play critical roles in post‐transcriptional gene regulation, including alternative splicing, mRNA transport and localization, translation initiation, and stress granule formation.^[^ ^84^ ^]^ Emerging evidence, supported by G4‐specific antibody staining, fluorogenic probes, and high‐resolution techniques, has confirmed the cytoplasmic abundance, dynamic folding behavior, and regulatory complexity of RNA G4s in living cells.^[^ ^85^, ^86^ ^]^ These structures are highly responsive to cellular conditions, with increased formation observed following depletion of G4‐interacting proteins, and upon treatment with G4‐stabilizing ligands.^[^ ^85^ ^]^ Despite their biological significance, RNA G4s remain largely underexplored as targets for photoresponsive ligand development. This presents a compelling opportunity to design light‐activated ligands capable of selectively binding and modulating RNA G4s. Such tools could offer spatiotemporal precision in regulating RNA metabolism, particularly translation and degradation, with promising therapeutic applications in cancer, neurodegenerative diseases, and certain viral infections.
2. *Optimizing nuclear delivery*: Most G4‑targeted PSs rely on extended π‑conjugated, hydrophobic scaffolds to stack onto terminal G‑tetrads. Unfortunately, this hydrophobicity often sequesters them in subcellular organelles devoid of G4 DNA, rather than permitting access to their nuclear targets. Traditional strategies, such as appending cationic residues or employing aptamer carriers, are still far from being optimal.^[^ ^87^ ^]^ We believe that alternative approaches should be explored, for example, grafting cell‐penetrating or short nuclear localization signal (NLS) peptides, such as PKKKRKV or RRKRQR, directly onto the PS scaffold.^[^ ^88^ ^]^ These NLS tags would exploit importin‑mediated transport to ferry the conjugate through the nuclear pore complex and, once inside, a cleavable linker could release the free PS to engage its G4 target with minimal off‑target accumulation.
3. *Controlling photochemical pathways*: Mechanistic fine‑tuning of ROS generation is as crucial as optimizing delivery. Most G4‑targeted PSs follow a Type I mechanism,^[^ ^26^, ^27^, ^28^ ^]^ producing O_2_^•−^ and/or OH^•^ species, which tend to inflict indiscriminate DNA damage. In contrast, Type II PSs generate ^1^O_2_, which preferentially oxidizes G at G4 sites and thus enables site‑selective photodamage.^[^ ^62^ ^]^ Striking the right balance of ROS type and yield is therefore essential when designing next‑generation, G4‑selective PSs. To that end, rigorous mechanistic studies must compare the photophysical behaviour of PSs both free in solution and bound to G4 targets.^[^ ^26^, ^30^ ^]^ Indeed, PS‐biomolecule binding can potentially induce photophysical switching, shifting the dominant ROS pathway. Consequently, probing whether G4 engagement can flip a PS from a Type I profile to Type II, or vice versa, is of paramount importance. These experimental studies could be complemented by quantum‐mechanical calculations. Building an open, freely accessible database that lets researchers retrieve photophysical data on triplet‐state dynamics would clarify structure‐reactivity relationships and pave the way for the rational design of PSs with predictable photochemistry. Such insights would unlock the potential to engineer PSs that generate oxidative lesions at single‑base resolution within specific G4 structures, ultimately enabling bespoke phototherapeutic agents tailored to individual G4 sequences.
4. *Advancing photocaged ligands*: Photocaged G4 ligands offer an elegant “turn‑on” strategy, remaining inert until activated by light, but early designs typically rely on high‑energy UV irradiation, which affords poor tissue penetration and risks collateral phototoxicity. Next‑generation scaffolds must shift activation into the red‐NIR window, either by incorporating red‑absorbing caging groups (e.g., BODIPY or cyanine moieties) or by harnessing two‑photon excitation to uncage with NIR lasers, thereby enabling deeper tissue reach, minimizing off‑target damage, and enhancing in vivo applicability.^[^ ^89^, ^90^ ^]^
5. *Engineering next‑generation photochromic switches*: Among photochromes, azobenzenes hold tremendous promise for dynamically controlling G4 structure and function. However, current designs fall far short of ideal PSS compositions, suffer off‑target interactions of one isomer, and depend on UV/blue/green light for switching, limitations that hinder clinical translation. To overcome these challenges, future efforts should target genuine “ON/OFF” behavior, achieving a PSS bias of ≥90 percent conversion to the active isomer within seconds while maintaining the inactive form fully off. Equally critical is red‐near‑infrared responsiveness, which can be achieved through ad‐hoc chemical substitutions or bridged azobenzene motifs.^[^ ^90^, ^91^ ^]^ Incorporating fluorogenic moieties that light up upon isomerization would further enable simultaneous diagnostic imaging and real‑time tracking of intracellular distribution.^[^ ^92^ ^]^ Finally, high‑resolution crystal or cryo‑EM structures of both isomeric forms bound to G4 targets are indispensable for elucidating distinct binding modes and guiding rational improvements in affinity and selectivity. Together, these advances will pave the way for reversible, light‑controlled G4 therapeutics with unparalleled precision.

Overall, light‑powered approaches for G4 targeting hold tremendous promise for precision phototherapy and photopharmacology. By overcoming delivery challenges, mastering photochemical pathways, and advancing both “caged” and reversible photoactive scaffolds, ideally guided by rigorous mechanistic studies and AI‑driven ligand design, we can unlock deep‑tissue activation strategies that propel G4‑focused agents into clinical evaluation. The next era of G4 phototherapeutics will be defined by agents that not only bind with molecular exactitude but also activate at the right place, the right time, and with minimal off‑target effects, ushering in truly precision medicine powered by light.

## Conflict of Interest

The authors declare no conflict of interest.

## Data Availability

Data sharing is not applicable to this article as no new data were created or analyzed in this study.
